# Supplementary material for: SWITCH 1/DYAD is a WINGS APART-LIKE antagonist that maintains sister chromatid cohesion in meiosis
Source: Nat Commun. 2019 Apr 15;10:1755. doi: 10.1038/s41467-019-09759-w (PMC6465247; doi:10.1038/s41467-019-09759-w)
Supplement: Supplementary file 1 — Supplementary Information [file 41467_2019_9759_MOESM1_ESM.pdf]

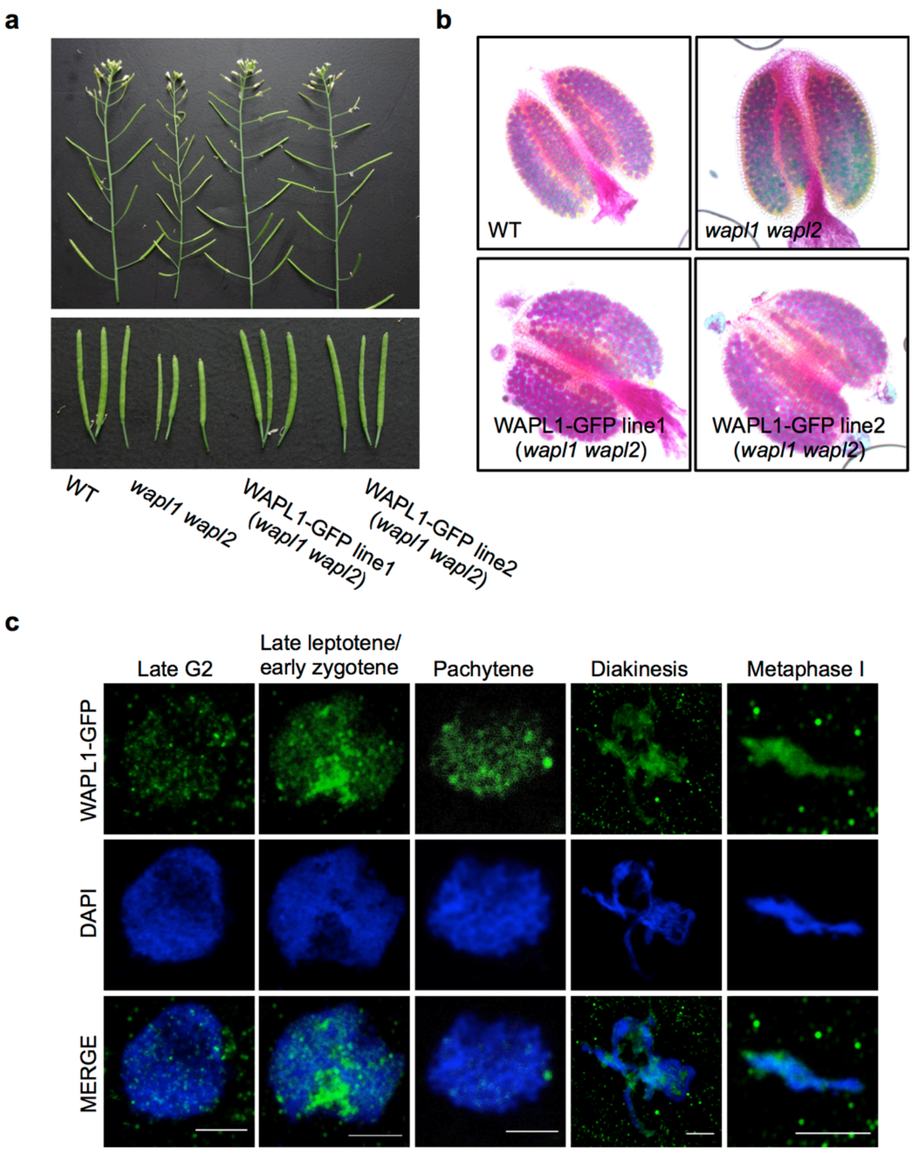

Supplementary Figure 1

WAPL1-GFP is functional and accumulates on chromatin from late leptotene/early zygotene till metaphase I. (a) Main branches (upper panel) and siliques (lower panel) of the wildtype (WT), *wapl1 wapl2*, and two lines expressing WAPL1-GFP in a *wapl1 wapl2* mutant background. (b) Peterson staining of anthers in the wildtype (WT), *wapl1 wapl2* mutants, and two WAPL1-GFP lines. Blue staining indicates dead pollen. (c)

Immunolocalization of WAPL1-GFP during meiosis I of male meiocytes. Anti-GFP antibody was used for detecting WAPL1-GFP. Bar: 5 μm.

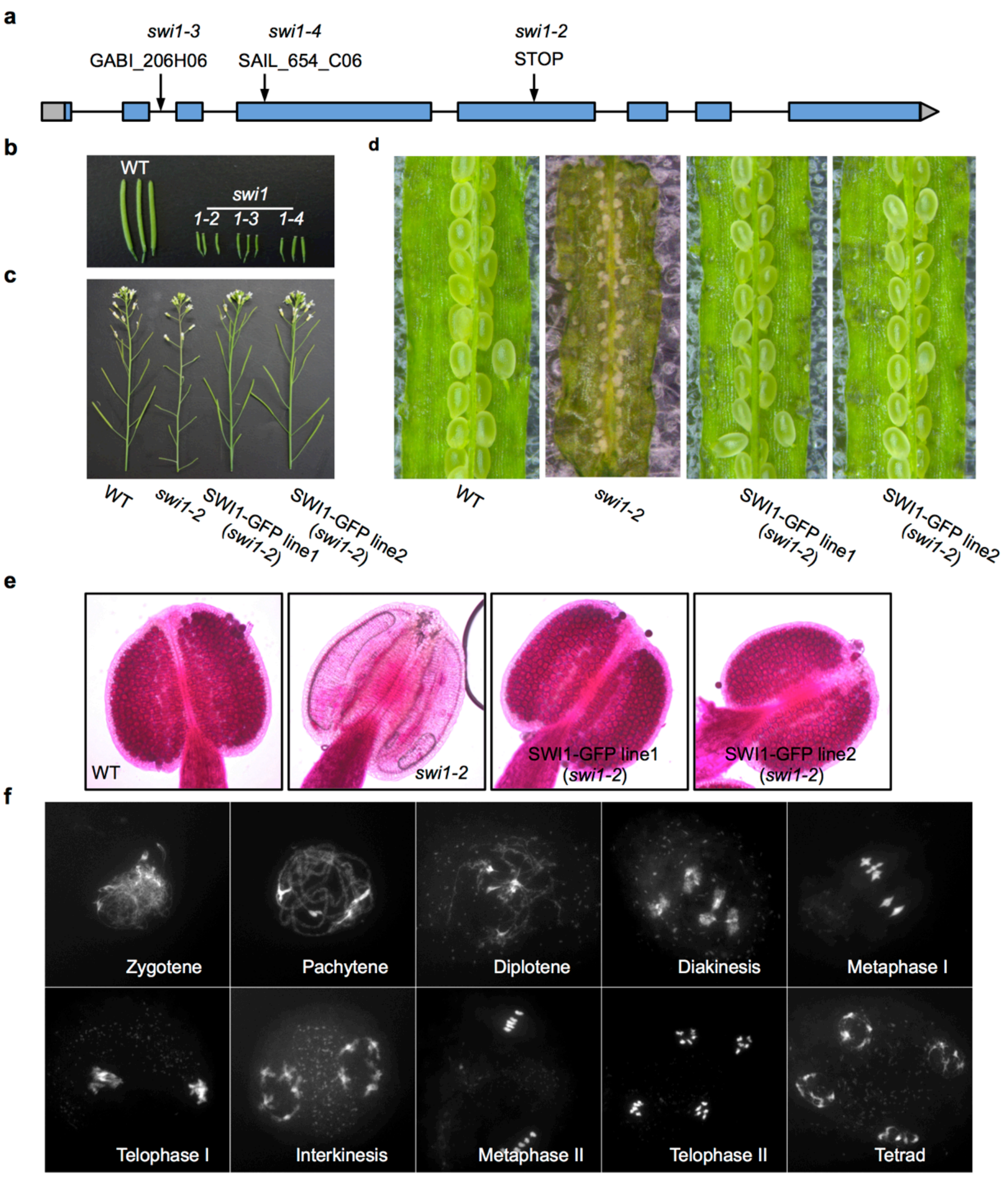

Supplementary Figure 2

SWI1-GFP fully complements the meiotic defects in *swi1* mutants. (a) Scheme of the genomic region of SWI1. Arrows denote the position of T-DNA insertions (*swi1-3*, *swi1-4*) and of a premature stop codon (*swi1-2*). (b) Siliques of the wildtype (WT) and different *swi1* mutant alleles, which are completely sterile. (c) The main branches of the wildtype, *swi1-2* and two *SWI1-GFP* lines. (d) Seed sets in siliques of the wildtype, two *SWI1-GFP* lines

and the *swi1-2* mutant. (e) Peterson staining of pollen for the wildtype, *swi1-2* and *SWI1-GFP* lines. No pollen was found in the *swi1-2* mutants. Blue staining indicates dead pollen. (f) Chromosome spread analysis of male meiocytes in *SWI1-GFP* line #2 (*swi1-2*) shows a wild-type meiotic program.

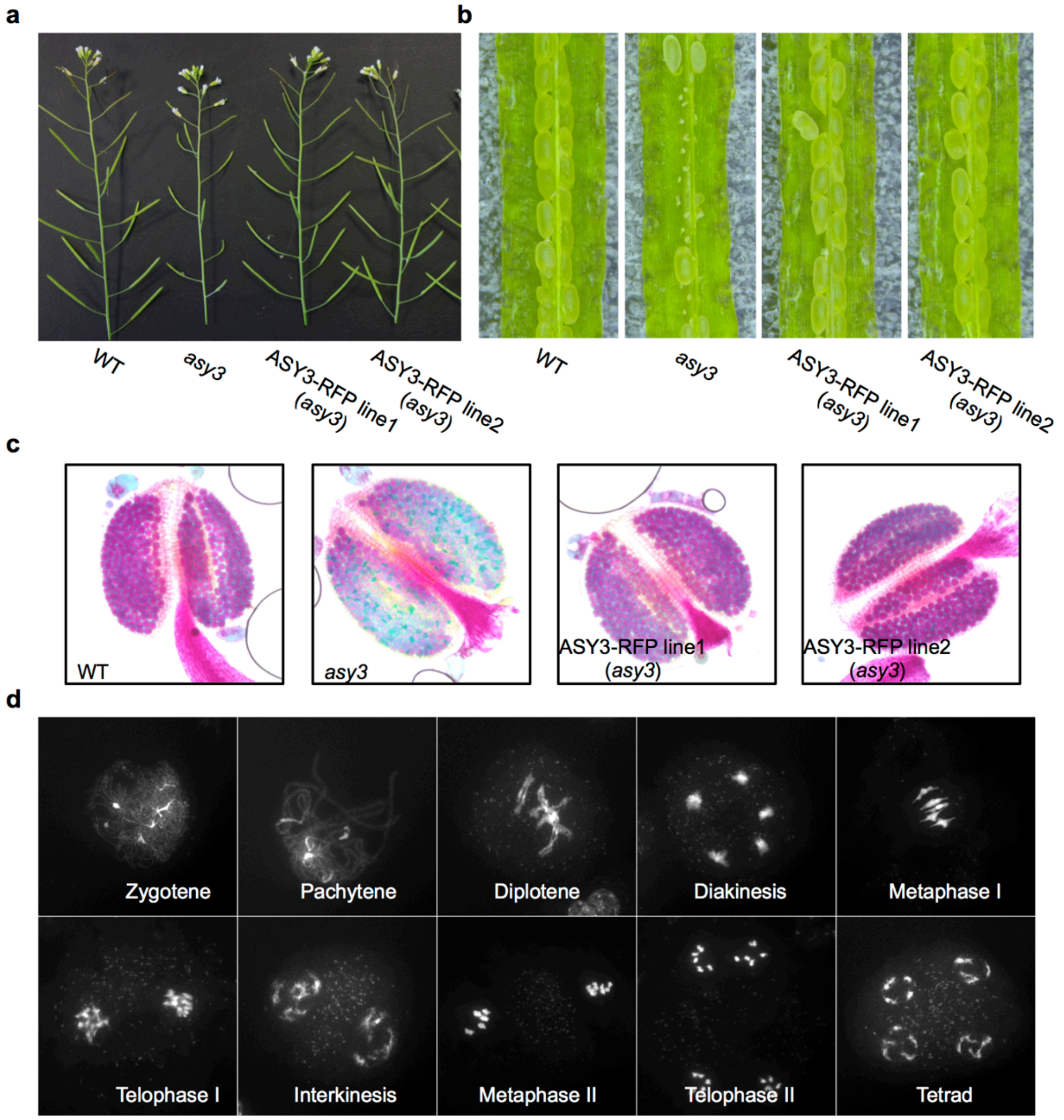

Supplementary Figure 3

ASY3-RFP fully complements the meiotic defects in *asy3* mutants. (a) The main branches of the wildtype (WT) and two *ASY3-RFP* lines. (b) Seed sets in siliques of the wildtype (WT) and two *ASY3-RFP* complementary lines. (c) Peterson staining of pollens for the wildtype (WT), *asy3* and *ASY3-RFP* lines. (d) Chromosome spread analysis of male meiocytes in *ASY3-RFP* line #1 (*asy3*) reveals a normal meiotic program.

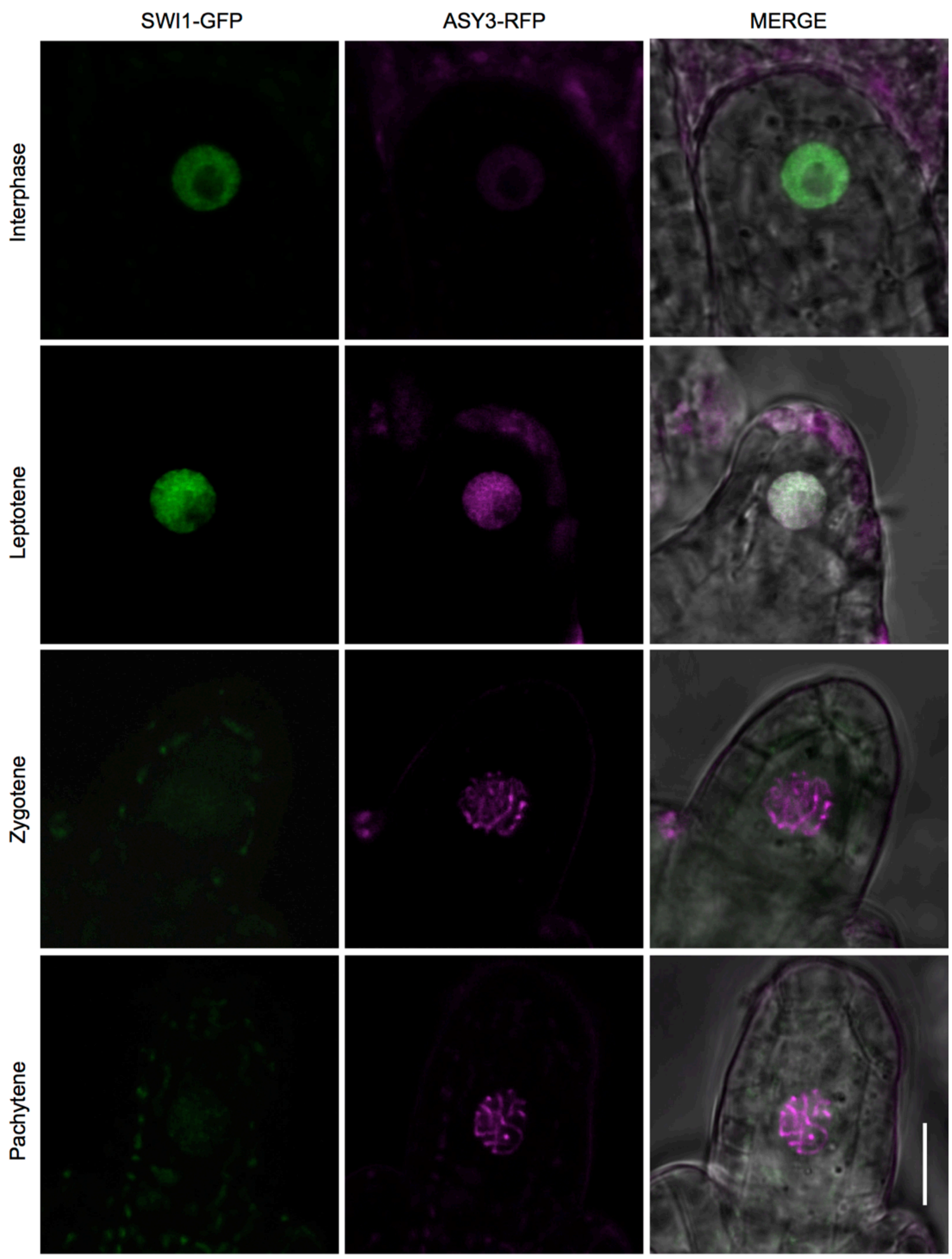

Supplementary Figure 4

Co-localization of SWI1 with ASY3 in female meiocytes. Co-localization analysis of SWI1-GFP (green) with ASY3-RFP (red) during interphase and prophase I in female meiocytes by using confocal laser scanning microscopy. Bar: 10 μm.

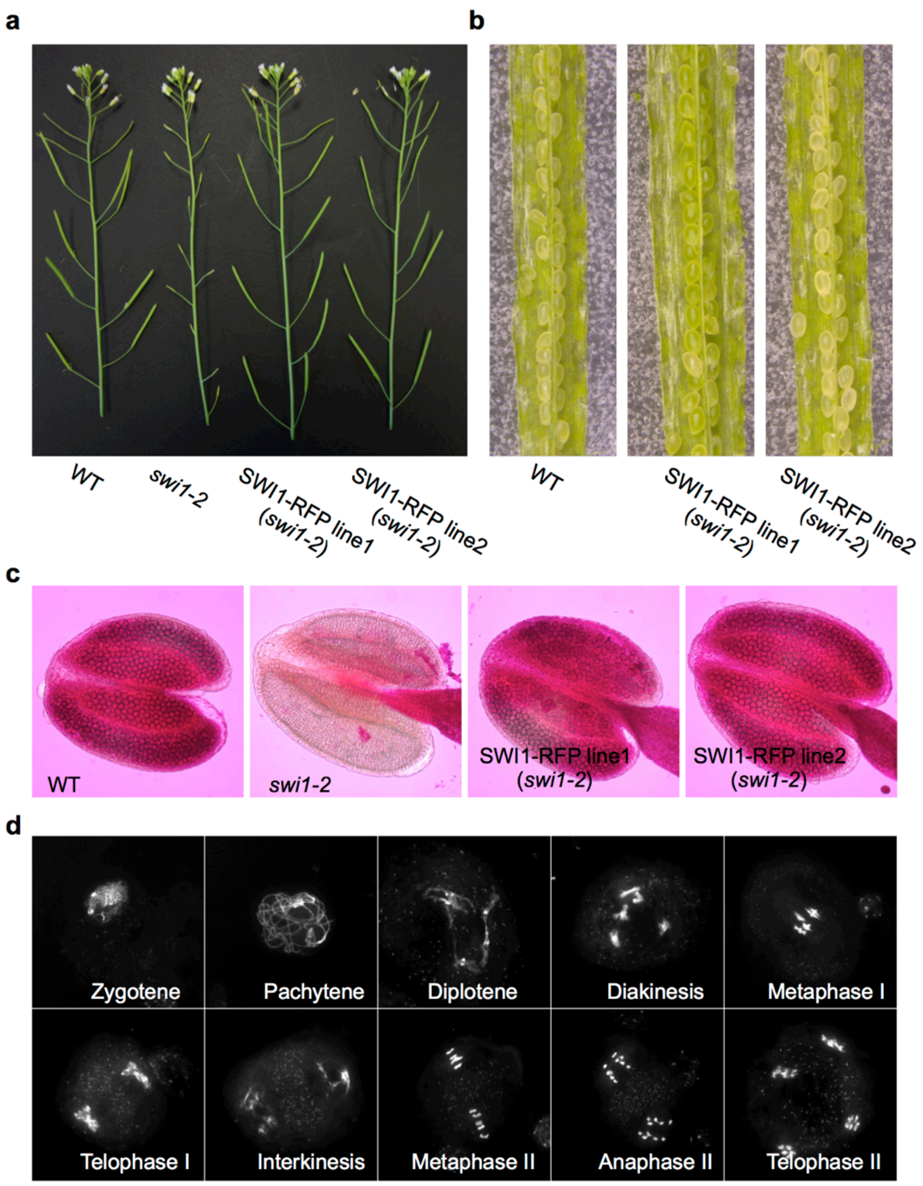

Supplementary Figure 5

SWI1-RFP fully complements the meiotic defects in *swi1* mutants. (a) Main branches of the wildtype (WT), *swi1-2* and two *SWI1-RFP* lines. (b) Seed sets in siliques of the wildtype (WT) and two *SWI1-RFP* lines. (c) Peterson staining of anthers for the wildtype (WT), *swi1-2* and *SWI1-GFP* lines. (d) Chromosome spread analysis of male meiocytes in *SWI1-RFP* line #1 (*swi1-2*) reveals a wild-type meiotic program.

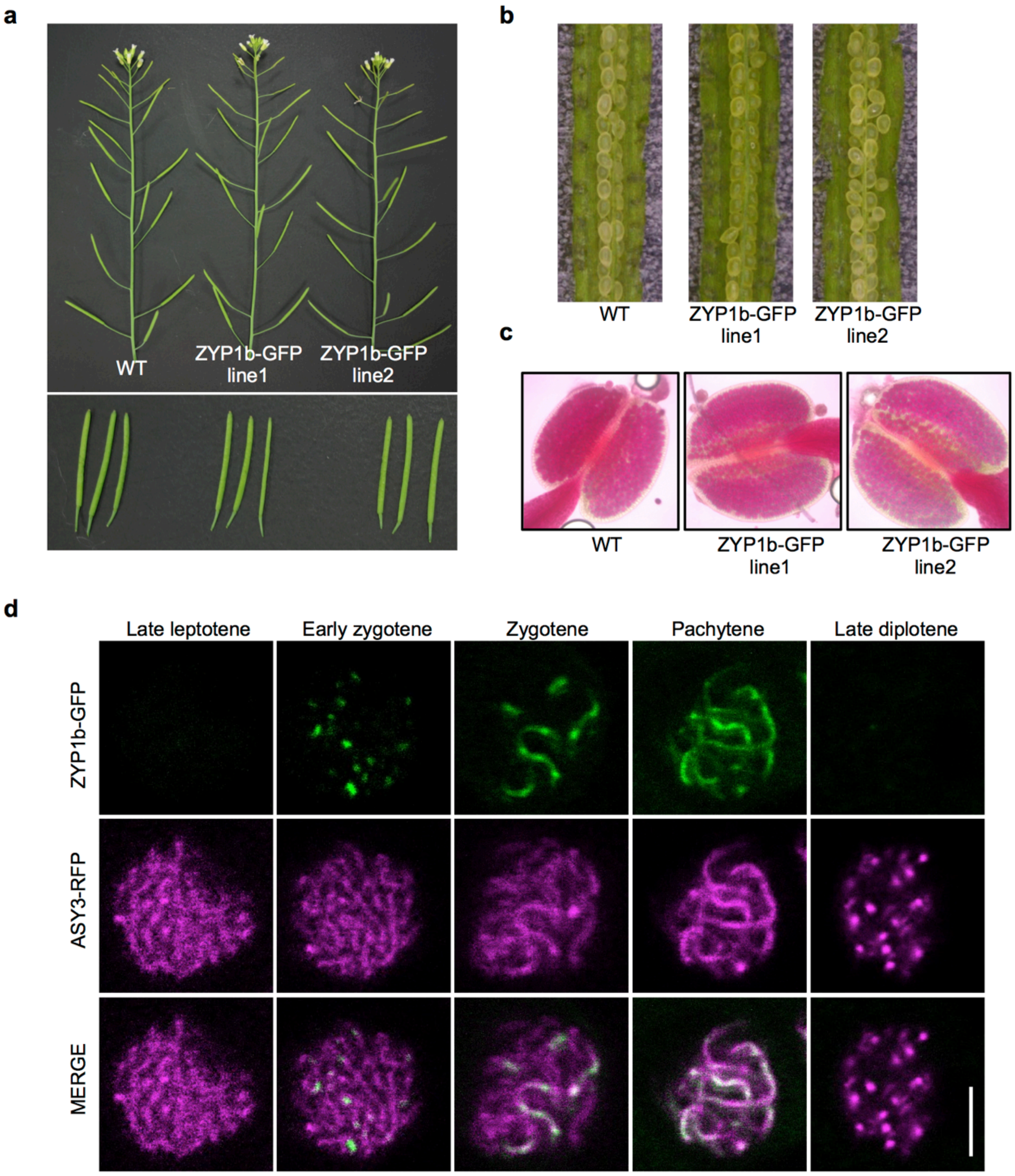

Supplementary Figure 6

ZYP1b-GFP is a good reporter for staging and has no dominant effect on plants. (a) Main branches of the wildtype (WT) and two *ZYP1b-GFP* lines in wildtype bacground. (b) Seed sets in siliques of the WT and two *ZYP1b-GFP* lines. (c) Peterson staining of anthers for the WT and and two *ZYP1b-GFP* lines. (d) Co-localization of ZYP1b-GFP with ASY3-RFP in the male

meiocytes of wildtype shows that ZYP1b-GFP specifically localizes to synaptic regions during prophase I. Bar: 5 µm.

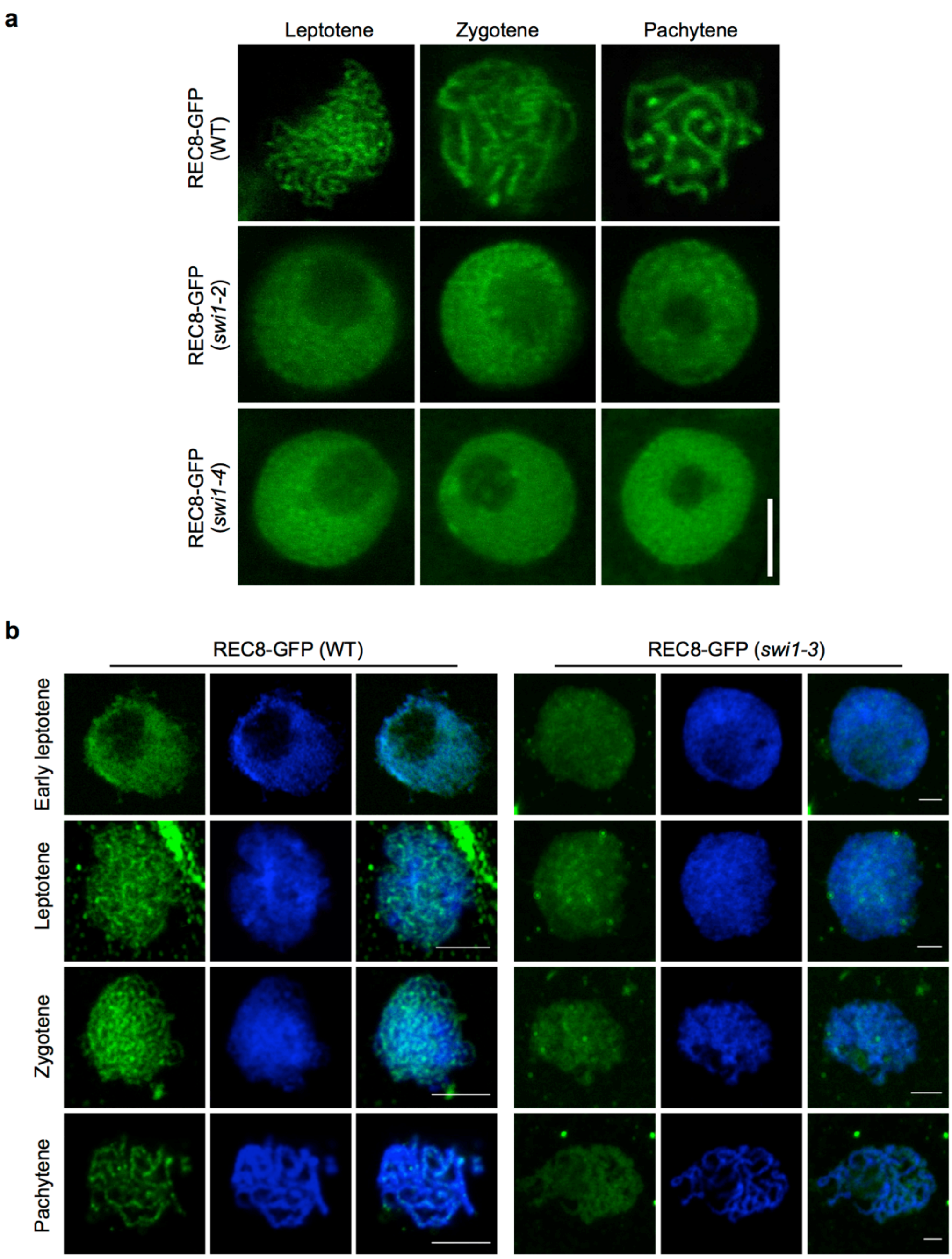

Supplementary Figure 7

Cohesion establishment is compromised in different *swi1* alleles. (a) Localization of REC8-GFP was analyzed by using laser confocal microscopy during prophase I of male meiocytes in the wildtype (WT), *swi1-2 and swi1-4*. Bar: 5 µm. (b) Immunolocalization of REC8-GFP in the male meiocytes of the

wildtype and *swi1-3* mutants during prophase I. Anti-GFP antibody was used for detecting REC8-GFP. Bar: 5 μm.

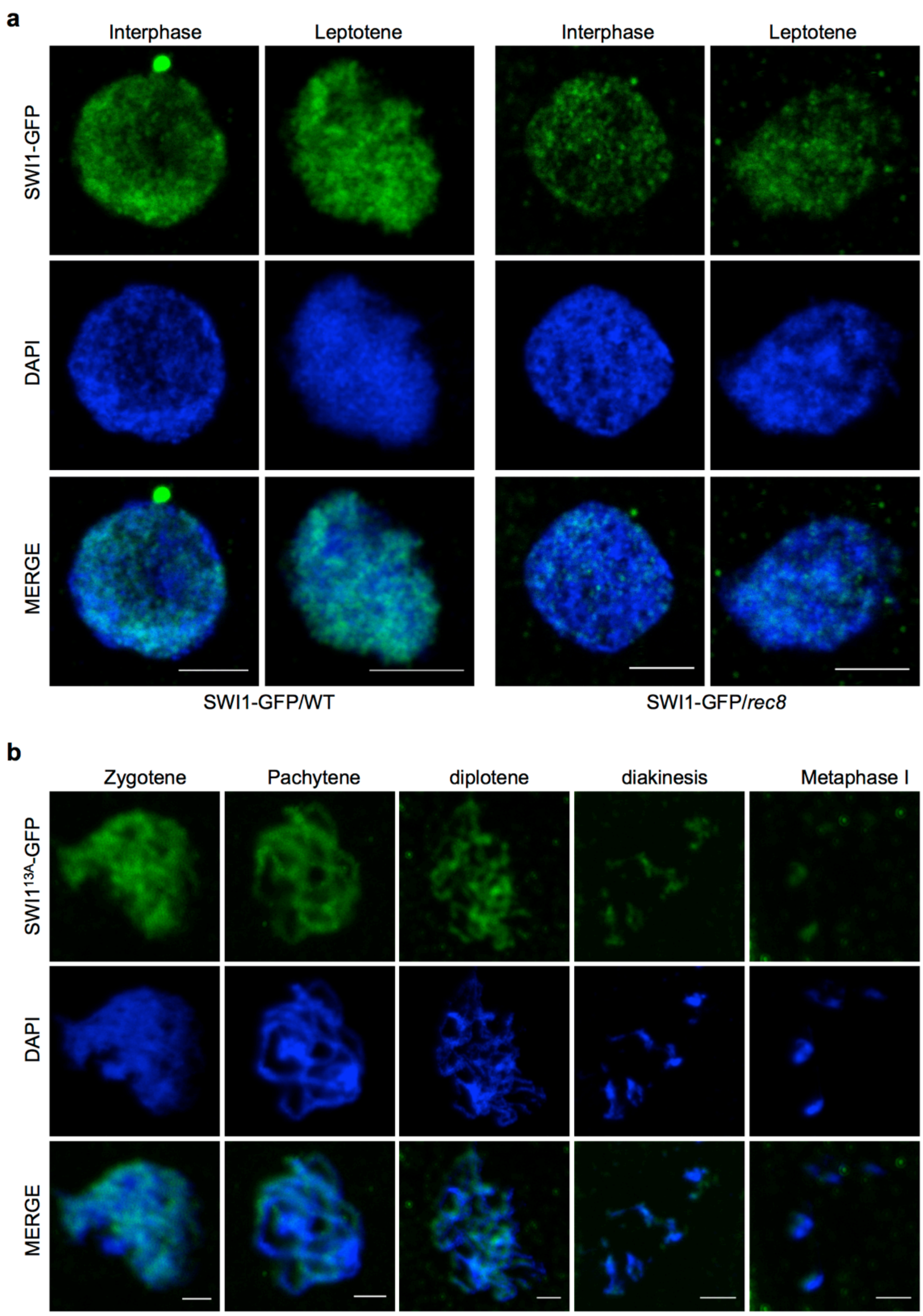

Supplementary Figure 8

Immunolocalization of SWI1-GFP and SWI1^{13A}-GFP. (a) Immunolocalization of SWI1-GFP in the wildtype (WT) and *rec8* mutants. Bar: 5 μm. (b) Immunolocalization of SWI1^{13A}-GFP in the wildtype. Bar: 5 μm. Anti-GFP antibody was used for detecting SWI1.

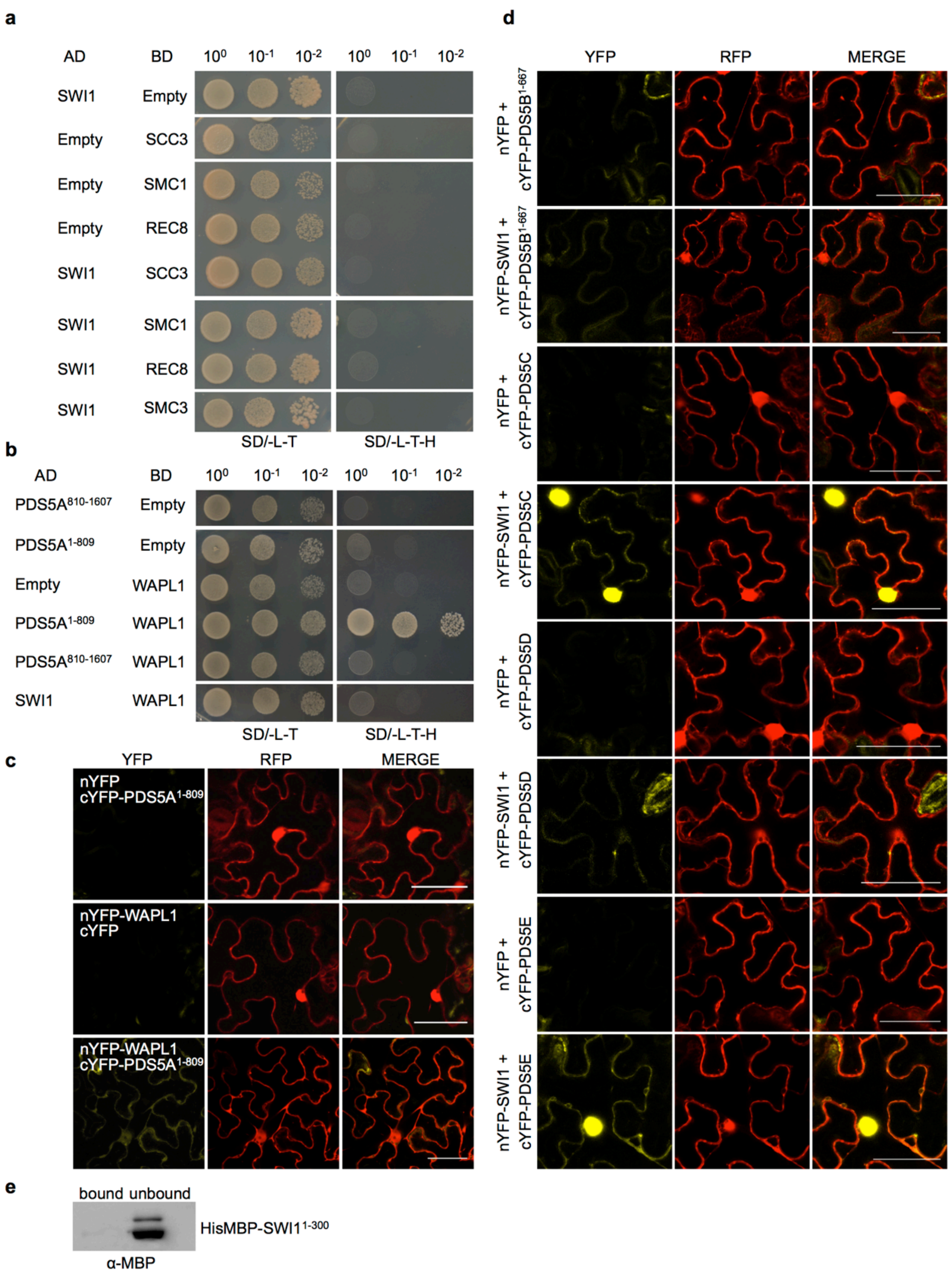

Supplementary Figure 9

Interaction analyses of SWI1, PDS5, and WAPL. (a) Yeast two-hybrid interaction assay of SWI1 with the core cohesin subunits SMC1, SMC3, REC8 and SCC3. (b) Yeast two-hybrid analysis of the interactions of WAPL1

with PDS5 and SWI1. (c) BiFC interaction assay of WAPL1 with PDS5A. Bar: 50 µm. (d) BiFC interaction assay of SWI1 with PDS5B, PDS5C, PDS5D, and PDS5E. (e) Immunoprecipitation for HisMBP-SWI1$^{1\text{-}300}$ only using GST binding beads showing no unspecific binding.

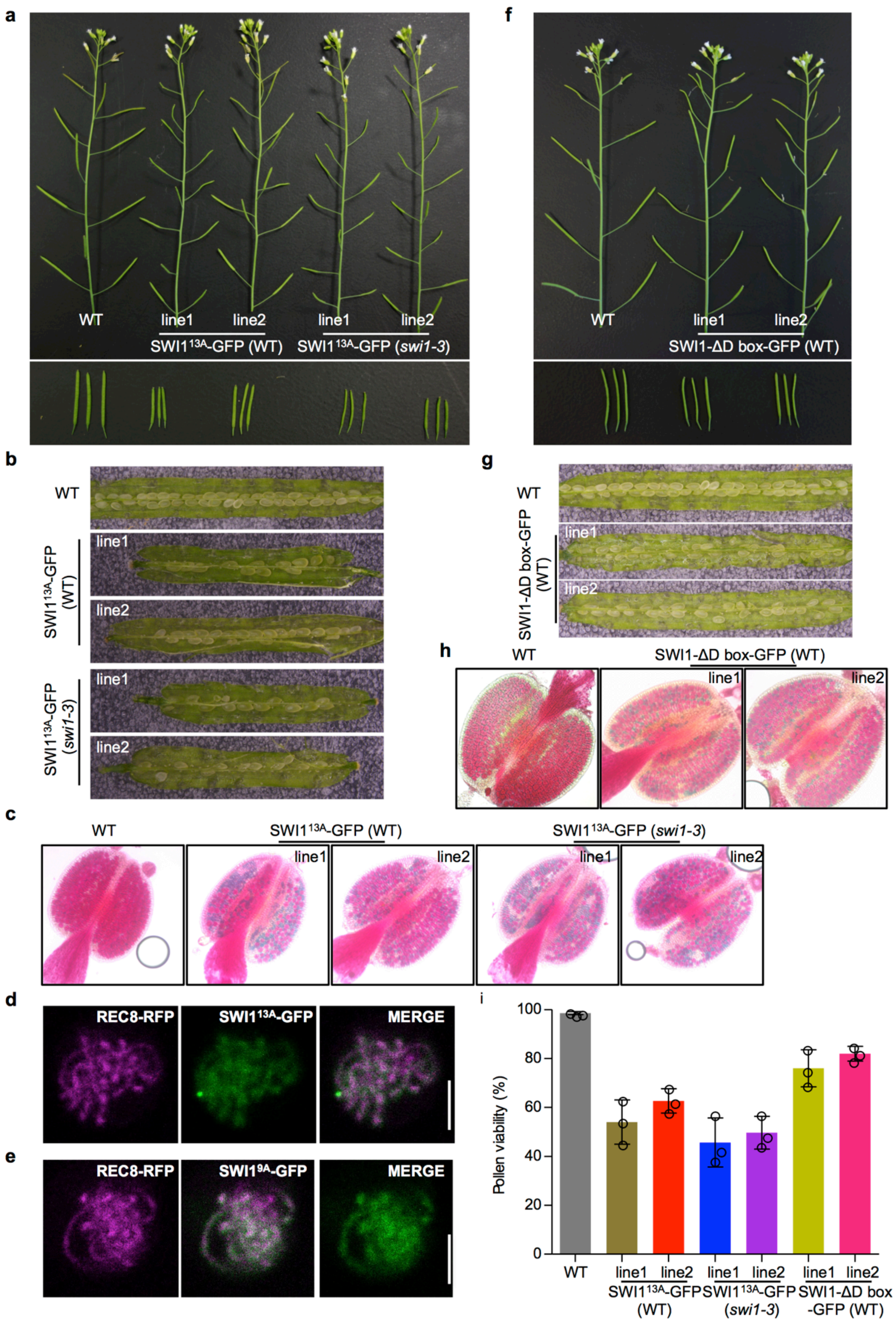

Supplementary Figure 10

Fertility of plants harboring the SWI1^{13A}-GFP and SWI1-ΔD box-GFP constructs. (a) Main branches (upper panel) and siliques (lower panel) of

the wildtype (WT) and two *SWI1^{13A}-GFP* lines in both WT and *swi1-3* muatnt background. (b) Seed sets in siliques of the WT and two *SWI1^{13A}-GFP* lines in both WT and *swi1-3* muatnt background. (c) Peterson staining of anthers for the WT and and two *SWI1^{13A}-GFP* lines in both WT and *swi1-3* muatnt background. (d, e) Co-localization of REC8-RFP and SWI1^{13A}-GFP (d) and SWI1^{9A}-GFP (e) in the male meiocytes of *swi1-3* mutant at pachytene. Bar: 5 µm. (f) Main branches (upper panel) and siliques (lower panel) of the wildtype (WT) and two *SWI1-ΔD box-GFP* lines in WT background. (g) Seed sets in siliques of the WT and two *SWI1-ΔD box-GFP* lines in WT background. (h) Peterson staining of anthers for the WT and and two *SWI1-ΔD box-GFP* lines in WT background. (i) Quantification of the pollen viability for the plants shown in (a) and (f). Error bars represent standard deviations.

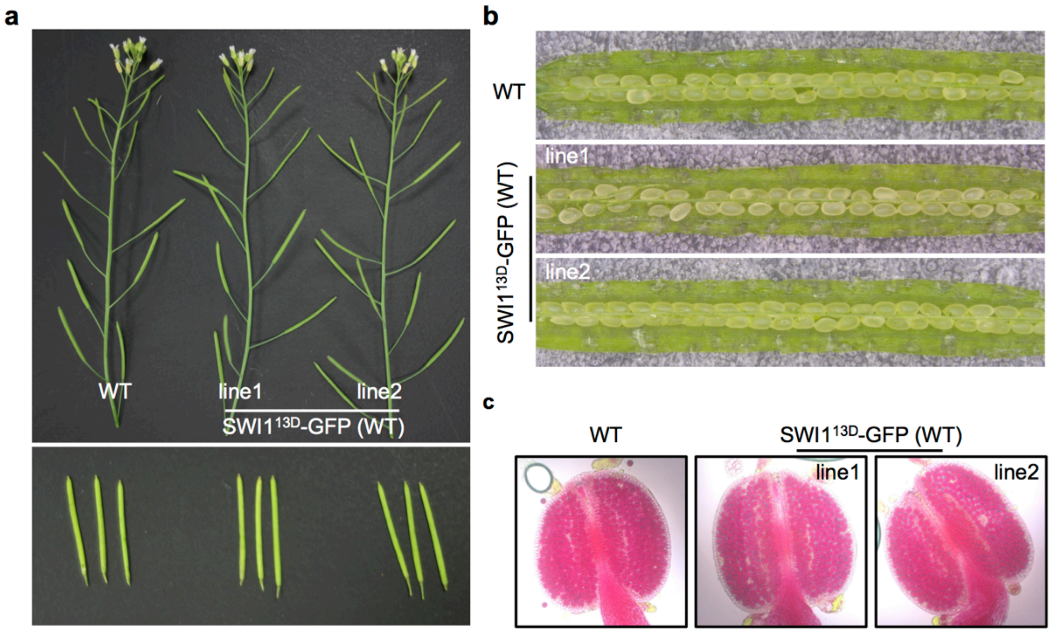

Supplementary Figure 11

Fertility of plants harboring the SWI1^{13D}-GFP construct. (a) Main branches (upper panel) and siliques (lower panel) of the wildtype (WT) and two *SWI1^{13D}-GFP* lines in wildtype (WT) background. (b) Seed sets in siliques of the WT and two *SWI1^{13D}-GFP* lines. (c) Peterson staining of anthers for the WT and and two *SWI1^{13D}-GFP* lines.

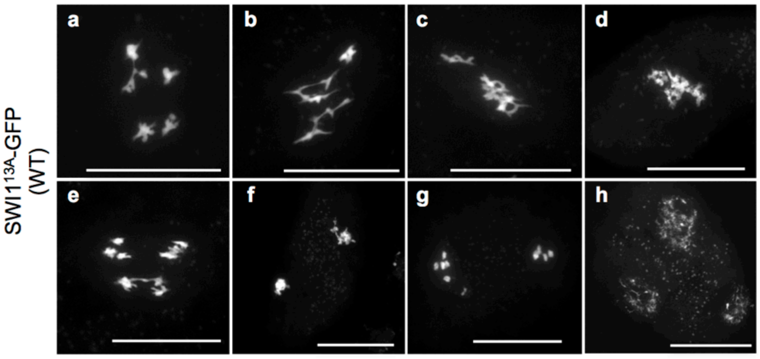

Supplementary Figure 12

Chromosome spread analysis of male meiocytes in *SWI1*^{13A}*-GFP*/WT plants. (a, b) diakinesis-like stage; (c, d) metaphase I-like stage; (e) anaphase I; (f, g) late telophase I or interkinesis; (h) Tetrad-like stage. Bar: 20 µm.

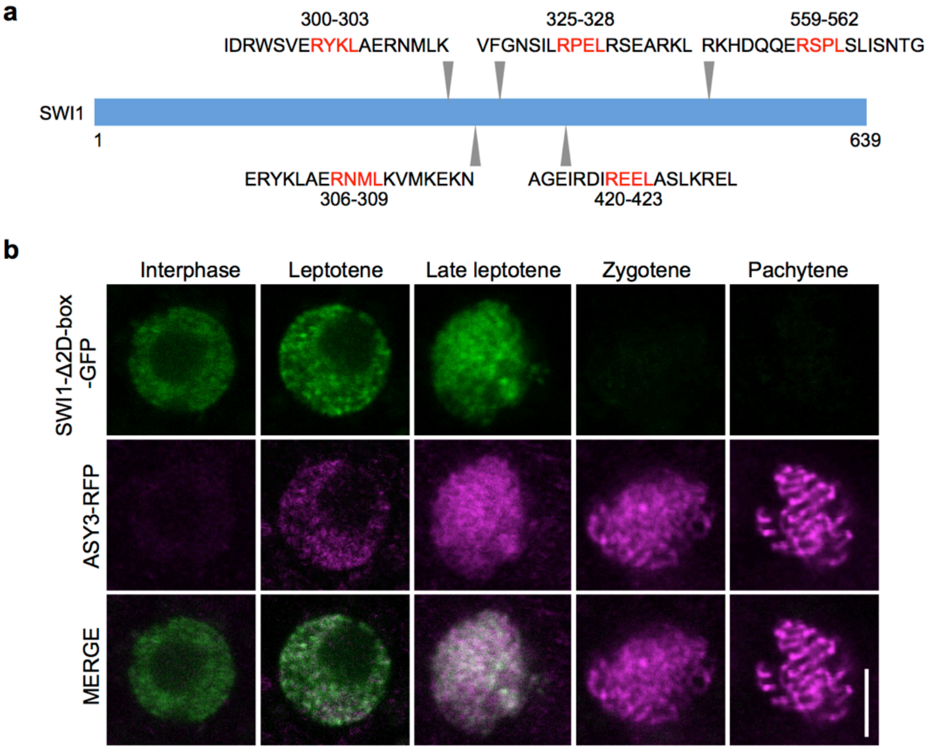

Supplementary Figure 13

Mutation of two conserved destruction box (D-box) has no impact on the localization pattern of SWI1. (a) Scheme of SWI1 coding region. Arrowheads indicate the positions of five putative D-boxes. (b) Localization analysis of SWI1-Δ2D-box-GFP (306-309 and 559-562) in the male meiocytes during prophase I. Bar: 5 μm.

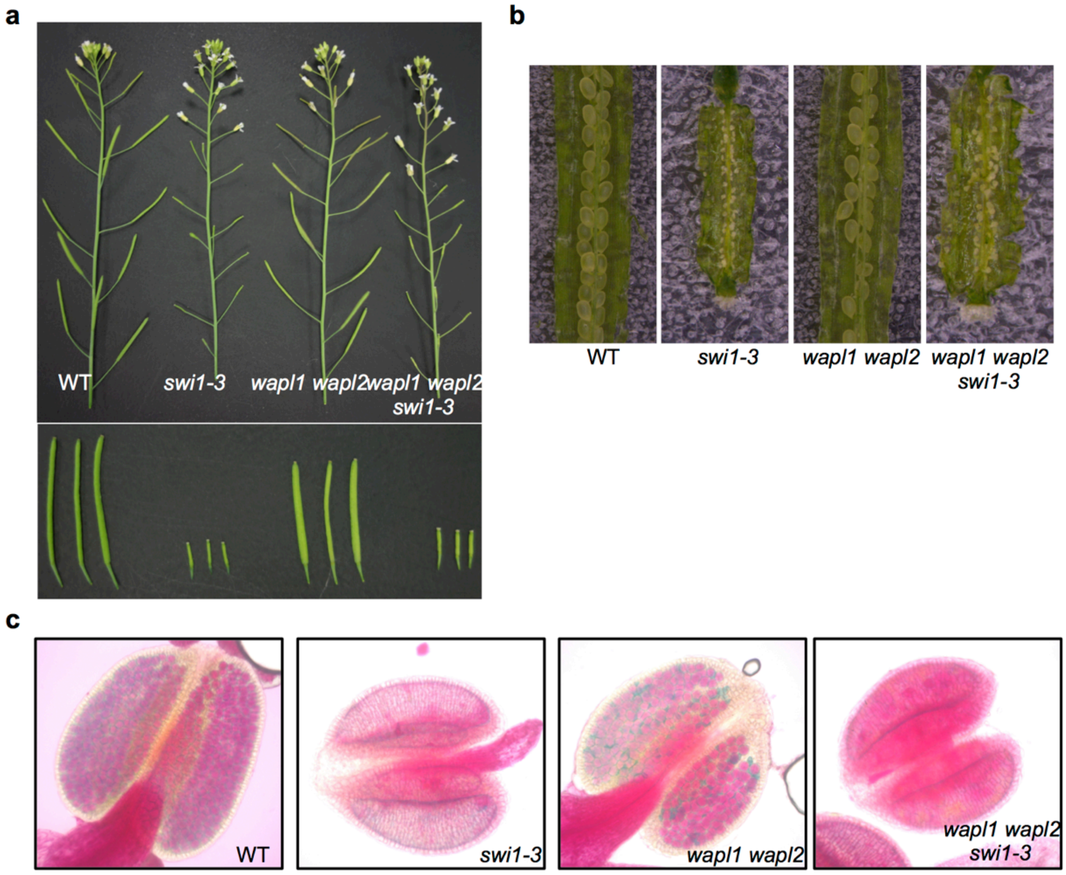

Supplementary Figure 14

The absence of *WAPL1 WAPL2* does not restore the fertility of *swi1-3* mutants. (a) Main branches (upper panel) and siliques (lower panel) of the wildtype (WT), *swi1-3*, *wapl1 wapl2* and *wapl1 wapl2 swi1-3* mutants. (b) Seed sets in siliques of the wildtype (WT), *swi1-3*, *wapl1 wapl2* and *wapl1 wapl2 swi1-3* mutants. (c) Peterson staining of anthers for the wildtype (WT), *swi1-3*, *wapl1 wapl2* and *wapl1 wapl2 swi1-3* mutants.

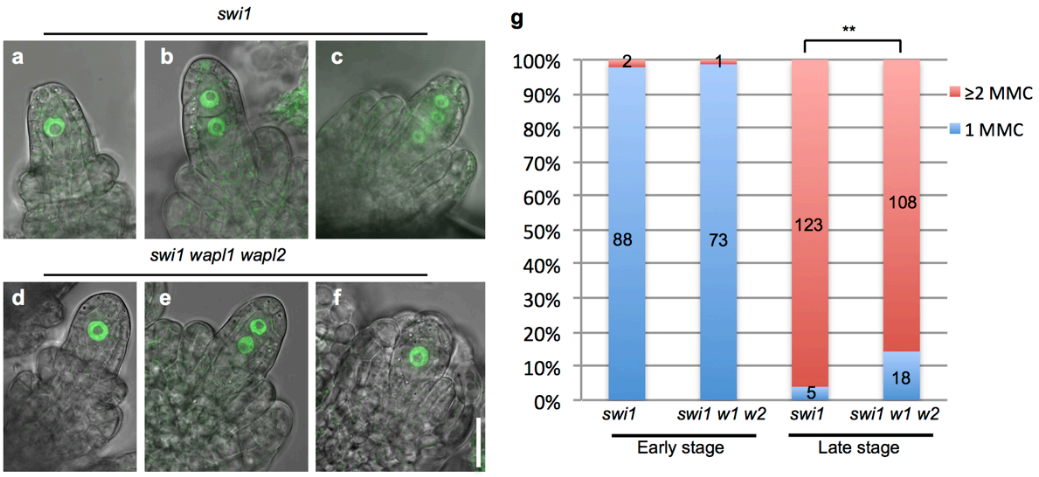

Supplementary Figure 15

The formation of single megaspore mother cell (MMC) in *swi1* mutants is partially rescued by depletion of WAPL. REC8-GFP was used as a maker for the counting of MMC. (a, d) Early premeiotic ovule with short integument primordia harboring one MMC. (b, e) Further developed ovules with elongated integuments containing two MMCs. (c) Older ovules than shown in (b) encompassing four MMCs. (f) Ovule at the similar stages as shown in (b, e) with only one MMC. Bar: 20 µm. (g) Statistical analysis of the number of MMCs at early (as in a, d) and late stages (as in b, c, e, f) in *swi1* and *swi1 wapl1 wapl2* (*swi1 w1 w2*) mutants. The numbers on the columns denote the amount of ovules counted. Asterisks indicate the significant difference (Chi-squared test, P< 0.01).

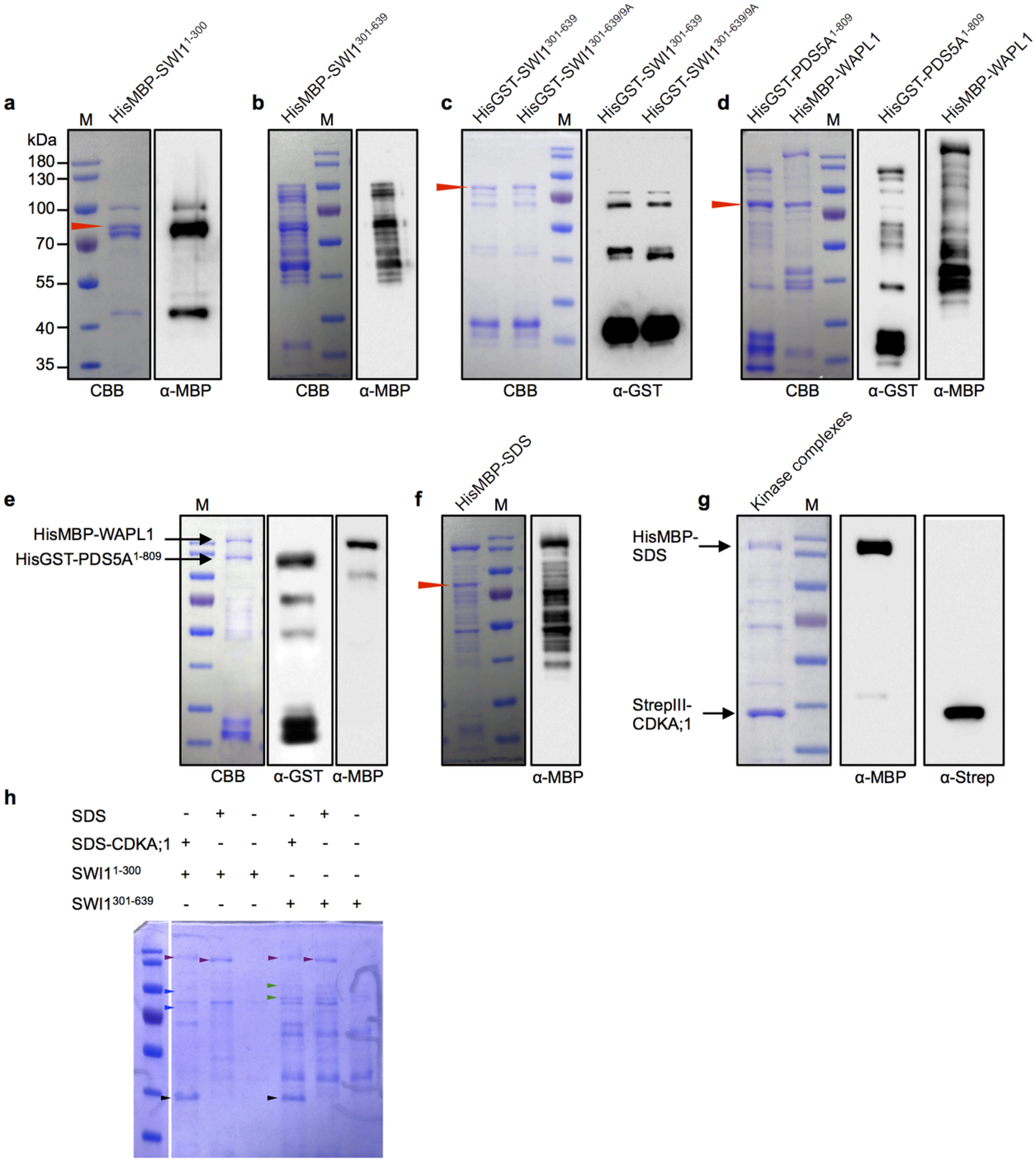

Supplementary Figure 16

CBB stained gels of all purified proteins from *Escherichia Coli* used in this research. (a-c) CBB staining and western blot confirmation of purified HisMBP-SWI1$^{1\text{-}300}$ (a), HisMBP-SWI1$^{301\text{-}639}$ (b), HisGST-SWI1$^{301\text{-}639}$ and HisMBP-SWI1$^{301\text{-}639/9A}$ (c), HisMBP-PDS5A$^{1\text{-}809}$ and HisMBP-WAPL1 (d), HisMBP-PDS5A$^{1\text{-}809}$-HisMBP-WAPL1 heterodimers (e), HisMBP-SDS (f) and CDKA;1-SDS complexes (g) from *E.coli*. The arrowheads indicate the bands

of unspecific protein binding generally to Ni-NTA beads. (h) CBB staining of the proteins after kinase reaction of SWI1 with CDKA;1-SDS complexes. The purple, blue, green or black arrowheads denote the main bands of SDS, SWI1$^{1\text{-}300}$, SWI1$^{301\text{-}639}$ or CDKA;1 proteins, respectively.

Supplementary table 1. Phosphorylated sites in SWI1.

Experiments	Proteins	Positions	Amino acid	Localization probabilities	peptides and Phospho Probabilities
replicate 1	SWI1 1-300; AT5G51330.1	22	S	0,99989	ISS(1)PSSPTLNVAVAHIR
	SWI1 1-300; AT5G51330.1	166	T	0,998469	REVVSQPAY(0.002)NT(0.998)R
	SWI1 301-639; AT5G51330.1	395	T	1,00000	EAGVKDPYWT(1)PPPGWK
	SWI1 301-639; AT5G51330.1	447	T	0,89016	KEEEELVIMT(0.11)T(0.89)PNSCVTSQNDNLMTPAK
	SWI1 301-639; AT5G51330.1	544	S	0,99992	VVNKGNQITES(1)PQNR
	SWI1 301-639; AT5G51330.1	560	S	1,00000	KHDQQERS(1)PLSLISNTGFR
	SWI1 301-639; AT5G51330.1	597	S	0.34000	ICRPVGMFAWPQLPALAAATDT(0.037)NAS(0.478)S(0.34)PS(0.117)HR
	SWI1 301-639; AT5G51330.1	606	S	1,00000	QAYPS(1)PFPVKPLAAK
replicate 2	SWI1 1-300; AT5G51330.1	22	S	0,99998	ISS(1)PSSPTLNVAVAHIR
	SWI1 1-300; AT5G51330.1	166	T	0,893899	REVVS(0.001)QPAY(0.106)NT(0.894)R
	SWI1 301-639; AT5G51330.1	395	T	1,00000	EAGVKDPYWT(1)PPPGWK
	SWI1 301-639; AT5G51330.1	447	T	0,60282	KEEEELVIMT(0.354)T(0.603)PNS(0.035)CVTSQNDNLMTPAK
	SWI1 301-639; AT5G51330.1	544	S	0,99997	VVNKGNQITES(1)PQNR
	SWI1 301-639; AT5G51330.1	560	S	0,98042	KHDQQERS(0.98)PLS(0.02)LISNTGFR
	SWI1 301-639; AT5G51330.1	597	S	0,70145	ICRPVGMFAWPQLPALAAATDTNAS(0.073)S(0.701)PS(0.225)HR
	SWI1 301-639; AT5G51330.1	606	S	0,99999	QAYPS(1)PFPVKPLAAK

Phosphorylated peptides of SWI1 were identified by mass spectrometry analysis after subjecting SWI1 to *in vitro* kinase assays with CDKA;1-SDS complexes, as shown in Fig. S9d. No phosphorylated peptides were found in reactions without CDKA;1. Results from two independent biological replicates are shown. Data are available via ProteomeXchange Consortium with the identifier PXD009959.

Supplementary Table 2. Primers used in this research.

Purpose	Prmer name	sequence
SWI1 reporter	gSWI1-F	TTGACATTGTGAGAGTAACG
	gSWI1-R	AACTAGTCTAGAGAACGGGT
	gSWI1-attB1-F	GGGGACAAGTTTGTACAAAAAAGCAGGCTTAGCACTTTATGGTTTTTCCG
	gSWI1-attB2SmaI-R	GGGGACCACTTTGTACAAGAAAGCTGGGTTTCACCCGGGAACGTTGAAGAGATTCTTGG
ASY3 reporter	gASY3-F	TTTGAGAACTCCACTTTACTGCGT
	gASY3-R	CTGCTACTATCTTGTCGTCTTCTC
	gASY3-attB1-F	GGGGACAAGTTTGTACAAAAAAGCAGGCTTAAAAACATTACTTCCCCTACCAAA
	gASY3-attB2-SmaI-R	GGGGACCACTTTGTACAAGAAAGCTGGGTTTCACCCGGGATCATCCCTCAAACATTCTGCGA
ZYP1b reporter	gZYP1b-F	GAAATCAGATGAGCCCTTCCTTAA
	gZYP1b-R	GGGAACTGACTTTGTGTGGTAGAC
	gZYP1b-attB1-F	GGGGACAAGTTTGTACAAAAAAGCAGGCTTAGAAATCAGATGAGCCCTTCC
	gZYP1b-attB2-R+T	GGGGACCACTTTGTACAAGAAAGCTGGGTTTTATCAATCAAATGCATAGGGATC
	gZYP1b-AscI-F	AAGCATGGCGCGCCGGTAATAAGAGAAGCGAGCA
	gZYP1b-AscI-R	AAGCATGGCGCGCCACCAAGACGAGATTCTTTCA
	GFP-AscI-F	AAGCATGGCGCGCCAGGTGGCGGTGGATCAGGCGG
	GFP-AscI-R	AAGCATGGCGCGCCCAGACCCTCCACCTCCCTTGT
Y2H	SMC1-EcoRI-F	CCGGAATTCATGCCTGCGATACAATCCCCATCG
	SMC1-SalI-R	ACGCGTCGACTCACGATTCTTGGTAGTTCCTAAGG
Y2H	SMC3-NcoI-F	CATGCCATGGGAATGTTTATCAAGCAGGTTATAATCG
	SMC3-BamHI-R	CGCGGATCCTCAGGTATCGTGGGACTGATCTTTC
Y2H	REC8-EcoRI-F	CCGGAATTCATGTTGAGACTGGAGAGTTTGATAG
	REC8-SalI-R	ACGCGTCGACTTACATGTTGGGTCCTCTTGCAATG
Y2H	SCC3-EcoRI-F	CCGGAATTCATGGAAGACAGTCCTCAAGGCCTTA
	SCC3-SalI-R	ACGCGTCGACTCAGTGTCCCTTGGACCGTTCACCC
Y2H	SWI1-EcoRI-F	CCGGAATTCATGAGTAGTACGATGTTCGTGAAAC
	SWI1-XhoI-R	CCGCTCGAGTCAAACGTTGAAGAGATTCTTGG
Y2H and protein expression	SWI1-attB1-F	GGGGACAAGTTTGTACAAAAAAGCAGGCTTCATGAGTAGTACGATGTTCGTGAAA
	SWI1-300aa-attB2-R	GGGGACCACTTTGTACAAGAAAGCTGGGTTTCACCTCTCAACAGACCATCTATCA
	SWI1-301aa-attB1-F	GGGGACAAGTTTGTACAAAAAAGCAGGCTTCTACAAACTAGCTGAGAGGAACATG
	SWI1-639aa-attB2-R	GGGGACCACTTTGTACAAGAAAGCTGGGTTTCAAACGTTGAAGAGATTCTTGGG
Y2H and protein expression	AtPDS5A 1-809aa-F	ATGGCTCAGAAGCCGGAGGAACAGTTGAAAG
	AtPDS5A 1-809aa-R	CTACTTAACCAACGTCTTGATCCCATATATCTTC
	AtPDS5A 810-1607aa-F	CTGAAGATATATGGGATCAAGACGTTGGTT
	AtPDS5A 810-1607aa-R	CTATATTGCTGTCCTCGAGATTGACTTACCCAC
	AtPDS5A 1-809-attB1 F	GGGGACAAGTTTGTACAAAAAAGCAGGCTTCATGGCTCAGAAGCCGGAGGAACAG
	AtPDS5A 1-809-attB2 R	GGGGACCACTTTGTACAAGAAAGCTGGGTTCTACTTAACCAACGTCTTGATCCCA
	AtPDS5A 810-1607-attB1 F	GGGGACAAGTTTGTACAAAAAAGCAGGCTTCCTGAAGATATATGGGATCAAGAC
	AtPDS5A 810-1607-attB2 R	GGGGACCACTTTGTACAAGAAAGCTGGGTTCTATATTGCTGTCCTCGAGATTGAC
Y2H and protein expression	WAPL1-CDS-F	ATGATAATTGTAAAACTAACGGCCAATCGC
	WAPL1-CDS-R	CTACGGTGATTTGCAGGATTCAATCACTCCC
	WAPL1-CDS-attB1-F	GGGGACAAGTTTGTACAAAAAAGCAGGCTTCATGATAATTGTAAAACTAACGGCC

	WAPL1-CDS-attB2-R	GGGGACCACTTTGTACAAGAAAGCTGGGTTCTACGGTGATTTGCAGGATTCAATC
Y2H	OsAM1-CDS F	ATGGACGCGGAGATGGCGGCTCCTGCGCTTG
	OsAM1-CDS R	TCAGCAGTAGGACGGAGTGGCCAGTGCCAGCTC
	OsAM1-attB1-F	GGGGACAAGTTTGTACAAAAAAGCAGGCTTCATGGACGCGGAGATGGCGGCTCC
	OsAM1-attB2-R	GGGGACCACTTTGTACAAGAAAGCTGGGTTTCAGCAGTAGGACGGAGTGGCCAG
	ZmAM1-CDS F	ATGGACGTAGAGACGGTGCAGGCGGGTCCTG
	ZmAM1-CDS R	TCAGCAGTAGGATGGAGTAGCCAGGGCCAGCTC
	ZmAM1-attB1-F	GGGGACAAGTTTGTACAAAAAAGCAGGCTTCATGGACGTAGAGACGGTGCAGGC
	ZmAM1-attB2-R	GGGGACCACTTTGTACAAGAAAGCTGGGTTTCAGCAGTAGGATGGAGTAGCCAG
Dephospho mutagenesis	SWI1 S22/25A-F	GCTCCGTCGGCTCCGACTTTGAATGgtaaactactga
	SWI1 S22/25-R	AGAGATTTTCCCGGCGGTGGTTTCT
	SWI1 S52A-F	GCTCCGGAAAATCTTAAATCGATTAGAG
	SWI1 S52-R	TCTCTGAGGAAGAATCGAAGCATCG
	SWI1 S173A-F	GCTCCGGAGGGAAAGTGCTCGTCTGAG
	SWI1 S173-R	AGCAGCGCGACAGAGACGAGTATTG
	SWI1 T242A-F	GCTAAACAAGAGGCAAAGGAGATAACTA
	SWI1 T242-R	GCCTCCTATTTCATTCCCATCATCA
	SWI1 S261A-F	GCTAGTACTGAGAGACTCGCTCAGAAAG
	SWI1 S261-R	TTCAATCAGCTTTCTCTTACGATT
	SWI1 T395A-F	GCTCCTCCACCTGGTTGGAAGCTTGGTG
	SWI1 T395-R	CCAGTAAGGATCTTTAACTCCTGCT
	SWI1 T447A-F	GCTCCTAATTCTTGTGTTACTAGTCAG
	SWI1 T447-R	AGTCATGATAACAAGCTCCTCCTCT
	SWI1 T461A-F	GCTCCAGCAAAGgtaagagctcgaaaca
	SWI1 T461-R	CATCAGATTATCATTCTGACTAGTA
	SWI1 T515A-F	GCTCCTTTGCTACTAGAGGATTCACCAC
	SWI1 T515-R	CTCTGTTGAGTCTGGCTTTTTAGGA
	SWI1 S522A-F	GCTCCACCAATACAGACACTAGAAGGAG
	SWI1 S522-R	ATCCTCTAGTAGCAAAGGTGTCTCT
	SWI1 S544A-F	GCTCCTCAAAACAGAGAAAAAGGAAGGA
	SWI1 S544-R	CTCTGTGATTTGGTTACCCTTGTTC
	SWI1 S560A-F	GCTCCACTTTCACTAATAAGCAACACTG
	SWI1 S560-R	TCTTTCTTGTTGATCATGCTTCCTT
	SWI1 S597A-F	GCTCCAAGTCACAGACAAGCCTACCCAT
	SWI1 S597-R	AGAAGCATTAGTATCAGTAGCAGCA
	SWI1 S606A-F	GCTCCTTTTCCAGTCAAGCCACTTGCAG
	SWI1 S606-R	TGGGTAGGCTTGTCTGTGACTTGGC
genotyping for *swi1-2*	SWI1-CAPS-F	AACAAGAGGCAAAGGAGATAAC
	SWI1-CAPS-R	TTTTCAGCAGATCAGCCGTAGA
genotyping for *swi1-3*	SAIL_654_C06 LP	ACTCATCACCGCTTGATTCTG
	SAIL_654_C06 RP	TGATACTGCACACGCAATCTC
genotyping for *swi1-4*	GABI_206H06 LP	CTCCCAGATTCATTAAATGCG
	GABI_206H06 RP	CTAGAAACCCAGAAACCCCAG
Phosphomimic mutagenesis	SWI1 S22/25D-F	GACCCGTCGGACCCGACTTTGAATGgtaaactactga
	SWI1 S22/25-R	AGAGATTTTCCCGGCGGTGGTTTCT
	SWI1 S52D-F	GACCCGGAAAATCTTAAATCGATTAGAG

Phosphomimic mutagenesis	SWI1 S52-R	TCTCTGAGGAAGAATCGAAGCATCG
	SWI1 S173D-F	GACCCGGAGGGAAAGTGCTCGTCTGAG
	SWI1 S173-R	AGCAGCGCGACAGAGACGAGTATTGTAC
	SWI1 T395D-F	GACCCTCCACCTGGTTGGAAGCTTGGTG
	SWI1 T395-R	CCAGTAAGGATCTTTAACTCCTGCT
	SWI1 T447D-F	GACCCTAATTCTTGTGTTACTAGTCAG
	SWI1 T447-R	AGTCATGATAACAAGCTCCTCCTCT
	SWI1 T461D-F	GACCCAGCAAAGgtaagagctcgaaaca
	SWI1 T461-R	CATCAGATTATCATTCTGACTAGTA
	SWI1 T515D-F	GACCCTTTGCTACTAGAGGATTCACCAC
	SWI1 T515-R	CTCTGTTGAGTCTGGCTTTTTAGGA
	SWI1 S544D-F	GACCCTCAAAACAGAGAAAAAGGAAGGA
	SWI1 S544-R	CTCTGTGATTTGGTTACCCTTGTTC
	SWI1 S560D-F	GACCCACTTTCACTAATAAGCAACACTG
	SWI1 S560-R	TCTTTCTTGTTGATCATGCTTCCTT
	SWI1 S597D-F	GACCCAAGTCACAGACAAGCCTACCCAT
	SWI1 S597-R	AGAAGCATTAGTATCAGTAGCAGCA
	SWI1 S522D-F	GACCCACCAATACAGACACTAGAAGGAG
	SWI1 S522-R	ATCCTCTAGTAGCAAAGGTGTCTCT
	SWI1 S606D-F	GACCCTTTTCCAGTCAAGCCACTTGCAG
	SWI1 S606-R	TGGGTAGGCTTGTCTGTGACTTGGC
BiFC constructs	AtPDS5A-attB1 F1	GGGGACAAGTTTGTACAAAAAAGCAGGCTTCATGGCTCAGAAGCCGGAGGAACAG
	AtPDS5A-attB4 R1-T 809aa	GGGGACAACTTTGTATAGAAAAGTTGGGTGCTTAACCAACGTCTTGATCCCA
	AtPDS5B-CDSattB1-F1	GGGGACAAGTTTGTACAAAAAAGCAGGCTTCATGGAGAAAACTCCGACGCAG
	AtPDS5B-CDSattB4-R1_T	GGGGACAACTTTGTATAGAAAAGTTGGGTGGCCACAAAGCTGATTCAAAAG
	AtPDS5C-CDSattB1-F	GGGGACAAGTTTGTACAAAAAAGCAGGCTTCATGTCGGATTCTGATAAAGAG
	AtPDS5C-CDSattB4-R_T	GGGGACAACTTTGTATAGAAAAGTTGGGTGTCGCTTCCTCTTCTTACCGG
	AtPDS5D-CDSattB1-F	GGGGACAAGTTTGTACAAAAAAGCAGGCTTCATGCAAAGTGCCCTAATTCCATC
	AtPDS5D-CDSattB4-R_T	GGGGACAACTTTGTATAGAAAAGTTGGGTGTGACTTTCTCTTCTCTTCATC
	AtPDS5E-CDSattB1-F	GGGGACAAGTTTGTACAAAAAAGCAGGCTTCATGGGTCCTCTTGTCGAAGCG
	AtPDS5E-CDSattB4-R_T	GGGGACAACTTTGTATAGAAAAGTTGGGTGTGGATCAACCTCAAGCACC
	AtSWI1-CDS-attB3-F	GGGGACAACTTTGTATAATAAAGTTGTAATGAGTAGTACGATGTTCGTGAAA
	AtSWI1-639-attB2-R-T	GGGGACCACTTTGTACAAGAAAGCTGGGTTAACGTTGAAGAGATTCTTGGG
	AtSWI1-300-attB2-R-T	GGGGACCACTTTGTACAAGAAAGCTGGGTTCCTCTCAACAGACCATCTATCA
	WAPL1-attB3-F	GGGGACAACTTTGTATAATAAAGTTGTAATGATAATTGTAAAACTAACGGCC
	WAPL1-attB2-R-T	GGGGACCACTTTGTACAAGAAAGCTGGGTTCGGTGATTTGCAGGATTCAATC
