# Supplementary material for: SWITCH 1/DYAD is a WINGS APART-LIKE antagonist that maintains sister chromatid cohesion in meiosis
Source: Nat Commun. 2019 Apr 15;10:1755. doi: 10.1038/s41467-019-09759-w (PMC6465247; doi:10.1038/s41467-019-09759-w)
Supplement: Supplementary file 3 — Description of Additional Supplementary Files [file 41467_2019_9759_MOESM3_ESM.pdf]

Description of Additional Supplementary Files

File Name: Supplementary Movie 1
Description: **Dynamics of REC8-GFP in wild-type plants.** Live cell imaging of REC8-GFP was performed in male meiocytes of wild-type plants. Movie starts at leptotene stage and runs for 25 h with scan intervals of 30 mins. Bar: 10 µm.

File Name: Supplementary Movie 2
Description: **Dynamics of REC8-GFP in wapl1 wapl2 mutants.** Live cell imaging of REC8-GFP was performed in male meiocytes of wapl1 wapl2 mutants. Movie starts at leptotene stage and runs for 25 h with scan intervals of 30 mins. Bar: 10 µm.

File Name: Supplementary Movie 3
Description: **Dynamics of REC8-GFP in swi1 mutants.** Live cell imaging of REC8-GFP was performed in male meiocytes of swi1 mutants. Movie starts at early zygotene-like stage and runs for 21 h with scan intervals of 15 mins. Bar: 10 µm.

File Name: Supplementary Movie 4
Description: **Dynamics of REC8-GFP in swi1 wapl1 wapl2 mutants.** Live cell imaging of REC8-GFP was performed in male meiocytes of swi1 wapl1 wapl2 mutants. Movie starts at early zygotene-like stage and runs for 21 h with scan intervals of 15 mins. Bar: 10 µm.

File Name: Supplementary Movie 5
Description: **Dynamics of REC8-GFP in wild-type plants.** Live cell imaging of REC8-GFP was performed in male meiocytes of wild-type plants. Movie starts at early leptotene stage and runs for 30 h with scan intervals of 15 mins. Bar: 10 µm.

File Name: Supplementary Movie 6
Description: **Dynamics of REC8-GFP in SWI113A-GFP/WT plants.** Live cell imaging of REC8-GFP was performed in male meiocytes of SWI113A-GFP/WT plants. Movie starts at early leptotene stage and runs for 35 h with scan intervals of 15 mins. Bar: 10 µm.
